# Lifestyle-Related Risk Factors and Primary Prevention Strategies for Cardiovascular Diseases in a Middle-Income Country: A Scoping Review and Implication for Future Research

**DOI:** 10.1007/s10935-024-00782-2

**Published:** 2024-06-05

**Authors:** Pragashini Raman, Yoganishalini Sagadevan, Sornavalli Dhanapalan, Brandon J. Fernandez, Sheng Yew Tan, Jamuna Rani Appalasamy, Amutha Ramadas

**Affiliations:** 1 Jeffrey Cheah School of Medicine and Health Sciences, Monash University Malaysia, Jalan Lagoon Selatan, 47500 Sunway City, Malaysia; 2 School of Pharmacy, Monash University Malaysia, Jalan Lagoon Selatan, 47500 Sunway City, Malaysia

**Keywords:** Cardiovascular disease, Metabolic syndrome, Risk factors, Primary prevention

## Abstract

Cardiovascular diseases are the leading cause of death in middle-income countries such as Malaysia. There is a significant gap in knowledge between cardiovascular disease-related risk assessments and interventions in the Malaysian population. In this scoping review, we have determined the status of cardiovascular research in Malaysia by prioritising lifestyle-related risk assessments and interventions. We searched five electronic databases (Ovid MEDLINE, Cochrane Central Register of Controlled Trials, APA PsychINFO, Embase and Scopus) to identify relevant research articles that had been published. The Joanna Briggs Institute and the preferred reporting items for systematic reviews and meta-analyses extension for scoping reviews served as a guide for the scoping review. Study selection was made using the Covidence platform, screened, and extracted. Thirty-one studies were included in this review. Studies reviewed reported a significant positive association between physical inactivity, smoking, poor dietary patterns, working hours, clustering of lifestyle risk, and cardiovascular disease risk. Most interventions focused on physical activity and a multimodal lifestyle approach, significantly improving primary and secondary cardiovascular disease-related outcomes. The findings suggest improving lifestyle-related risk assessments and interventions to prevent cardiovascular diseases in this population. It is unclear if these outcomes can translate to higher effectiveness in preventing cardiovascular disease. Nevertheless, intervention using the multifaceted lifestyle approach can improve cardiovascular disease-related outcomes.

**Supplementary Information:**

The online version contains supplementary material available at 10.1007/s10935-024-00782-2.

## Introduction

Cardiovascular diseases (CVDs) are a group of pathologies of blood vessels and cardiac nature. Ideally, CVDs can be classified into four major diagnostic categories: coronary artery disease, cerebrovascular disease, peripheral artery disease and aortic atherosclerosis (Lopez et al., 2022). CVDs have been identified as the leading cause of death worldwide. In 2019, approximately 17.8 million people died from CVDs, accounting for 32% of deaths globally (WHO, 2021). More significantly, the World Health Organization (WHO) reported that over 75% of CVD deaths occur in low- and middle-income countries (LMICs). This stems from the lack of primary health care services for early detection and interventions for people with predisposing risk factors for CVDs.

Although in 2022, Coronavirus Disease of 2019 (COVID-19) infection emerged as the primary cause of death in Malaysia, ischemic heart disease (IHD) remained a dangerous threat to the Malaysian population, with a prevalence of 13.7% (Department of Statistics Malaysia [DOSM], 2022). In the previous year, 2021, the prevalence of IHD in urban and rural areas was recorded as 17.2% and 16.5%, respectively (DOSM, 2021). The 2021 report further attested that males are more likely to succumb to these diseases than females, as the prevalence indicated that IHD caused death for 19.3% of males and only 13.4% of females (DOSM, 2021). Whereas another study in Malaysia reports the majority of individuals (69.9%) among the general population in Kuantan, Pahang recognise smoking as a risk factor for CVD-related deaths, followed by atrial fibrillation (AF) (57.7%), heart disease (54.1%), and obesity (53.8%) (Abdo Ahmed et al., 2020). Hence, lifestyle risk factors are critical for precipitating heart and cerebrovascular diseases (Lee et al., 2019).

The assessment of cardiovascular risk is critical for approaching the best intervention for the patient. The risk factors for CVDs are classified into modifiable and non-modifiable. The modifiable lifestyle risk factors include but are not limited to physical inactivity, poor dietary patterns, smoking, and alcohol intake, while the non-modifiable risk factors for CVD comprise increasing age, sex, family history, and ethnicity (Brown et al., 2022; IPH, 2019).

According to the Ministry of Health of Malaysia (MOH), the primary prevention of CVDs requires mass screening of individuals according to the components of the cardiovascular risk assessment, namely the history of smoking and vaping, blood pressure (BP), body mass index (BMI) and waist circumference (WC), lipid profile as well as blood glucose (MOH, 2017). Some lifestyle modifications recommended include a diet low in saturated fats, high in fibre, and low in sodium, regular exercise, smoking cessation, and maintaining an ideal body weight.

The existing evidence highlights the pressing issue of CVDs within the global and Malaysian contexts, underscoring their substantial burden on public health. With CVDs being the leading cause of mortality worldwide, particularly in LMICs, there's a critical need for comprehensive preventive measures to address this crisis. As evidenced by studies cited, the identification of lifestyle risk factors prevalent among Malaysian populations underscores the necessity for tailored interventions to mitigate CVD risks. Despite abundant evidence highlighting the prevalence and risk factors of CVD in Malaysia, there is a noticeable gap in examining the correlation between CVD-related risk assessments and interventions in Malaysia. Understanding this correlation is crucial for improving preventive strategies and interventions tailored to the specific needs of the Malaysian population.

Therefore, this scoping review provides an overview of conducted studies and knowledge regarding cardiovascular research in Malaysia by highlighting the existing studies and current understanding of risk factors and the effectiveness of interventions. In the broader field of prevention science, this scoping review aligns with the overarching goal of understanding and addressing the determinants of health outcomes through an evidence-based approach. By elucidating the correlation between CVD-related risk assessments and interventions in Malaysia, the study contributes to bridging a critical gap in preventive strategies. This effort is crucial for Malaysia and informing global efforts in preventive medicine and public health. By recognizing the significance of lifestyle factors in precipitating CVDs and proposing targeted interventions, we advocate for public health approaches to healthcare that ultimately improve population health outcomes. As such, we aim to enrich the field of prevention science by fostering a deeper understanding of the complex interaction between risk factors, interventions, and health outcomes in the context of CVDs.

## Methods

We utilised a scoping review design to comprehensively describe lifestyle-related risk and intervention strategies for CVD in Malaysia, a country battling a high burden of CVD, and to subsequently identify critical research and public health gaps in this area. This scoping review was guided using a combinatory approach based on methodological frameworks by the Joanna Briggs Institute (Peters et al., 2015), and guidelines developed by the preferred reporting items for systematic reviews and meta-analyses extension for scoping reviews (PRISMA-ScR) (Tricco et al., 2018). As only published data was used, ethical approval was not sought.

### Data Sources and Search Strategy

A systematic search was conducted in five electronic databases: Ovid MEDLINE, Cochrane Central Register of Controlled Trials, APA PsychINFO, Embase and Scopus. The search strategy consisted of the following combination of keywords: (“cardiovascular” OR “cardiovascular disease*” OR “cardiac” OR “coronary disease” OR “CVD” OR “CV” OR “Framingham” OR “heart disease* OR “metabolic syndrome”) AND (“lifestyle” OR “behavio* OR “nutrition” OR “diet*” OR “physical activit*” OR “exercise” OR “smoking” OR “tobacco” OR “sleep”) AND (Malaysia).

The search was limited to 1 January 2012 and 1 November 2023 and to adults (> 18 years) where possible. The restriction of the search to the years 2012–2023 aims to ensure the inclusion of the most recent evidence, thereby reflecting current trends in CVD prevention in Malaysia. Given the rapidly evolving landscape of healthcare and research, limiting the search to the last decade helps to incorporate the latest findings, providing a comprehensive overview of the current state of knowledge in this area. Additionally, limiting the search to adults serves two primary purposes: firstly, it allows for a focused examination of the demographic group most susceptible to CVD, and secondly, it helps reduce age-related bias in understanding both risk factors and intervention effects. By incorporating these restrictions, the review aims to provide a comprehensive and timely assessment of lifestyle risk factors and interventions for CVD prevention among Malaysian adults. No language restriction was imposed. Supplementary Table S1 demonstrates the search syntax employed for Ovid MEDLINE, which was similarly applied to the other four electronic databases.

### Study Selection

Study selection was conducted using the COVIDENCE platform (Veritas Health Innovation [VHI], 2022). After importing all searched articles, duplicates were eliminated, and the titles and abstracts were first screened, followed by full texts according to the predetermined eligibility criteria. Criteria for inclusion were peer-reviewed publications investigating lifestyle-related risk factors or interventions targeting lifestyle behaviors and health outcomes associated with cardiovascular diseases (CVDs) in Malaysian adults aged 18 and above. CVD was defined broadly to encompass both cardiac and vascular pathologies. Additionally, studies examining cumulative CVD risk factors, such as metabolic syndrome and changes in Framingham risk score, were considered. Clinical outcomes of interest included CVD incidence, cumulative CVD risk, and specific measures like blood pressure, biomarkers, and anthropometry. We excluded non-peer-reviewed publications, studies focusing on pharmacological interventions or clinical risk factors only, research conducted in populations with a history of CVD, and studies involving non-human subjects or methodological aspects.

Two authors independently performed screenings at both stages (YS and SD). Any disagreements that arose were arbitrated by a third author (SYT). Manual hand-searching of reference lists of included studies was also performed by two authors (PR and BJF) to seek articles not identified in the database searches.

### Data Extraction and Charting

This data extraction encompassed various aspects, including the publication year, study design, target population characteristics, study location and setting, the focus and duration of exposure or intervention, outcomes assessed, and primary findings. Subsequently, the selected studies were categorized based on their focus and outcomes to delineate the primary discussion areas of this review. This systematic approach to data charting ensured comprehensive coverage of relevant studies and facilitated the identification of key themes and findings for further analysis and discussion.

## Results

**Fig. 1:**
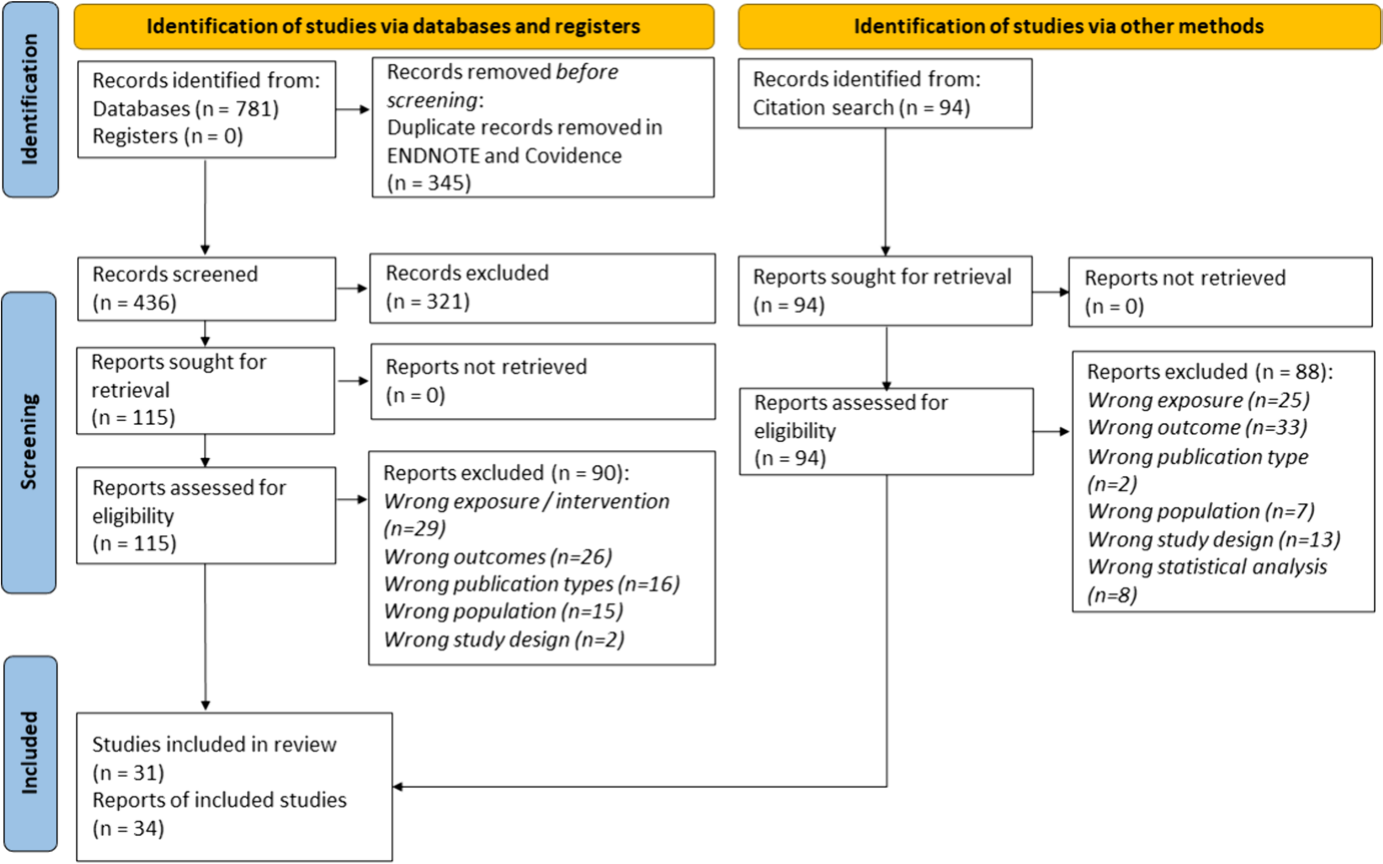
PRISMA 2020 flow chart showing the study selection process

Database searching resulted in 781 records. After removing 345 duplicates, 436 records were screened by title and abstract, of which 321 were excluded. A full-text review was performed for the remaining 115 articles, where 90 were excluded based on review eligibility criteria. Similarly, 94 records were identified using a manual search of the reference list, of which 88 articles were excluded after the full-text review. In the end, 34 articles representing 31 unique studies were included in this review. Figure 1 depicts the literature search and study selection process.

### Study Characteristics

Twenty-three studies focusing on risk factors assessments (Table 1) and eight on lifestyle interventions (Table 2) for CVD were included in this review. Most studies that explored lifestyle risk factors were cross-sectional (n = 19) and were conducted in the community (n = 11) or hospital/clinic (n = 7) (Table 3). While seven studies included participants from various parts of the country, the majority focused on selected states (n = 16). Physical activity was investigated as one of the exposures in the majority of the studies (n = 13), followed by 12 that reported on various aspects of diet.

**Table 1:** Studies reported on lifestyle risk factors for cardiovascular diseases among Malaysian adults (n = 23)

Study, study design, sample size	Target population Mean age ± SD (years)	Location	Study setting	Lifestyle risk factors	CVD-related outcomes	Main findings
Yeow et al. (2012) Cross-sectional N = 4341	Adults MetS: 52.7 ± 12.8 No MetS: 44.2 ± 14.7	Nationwide	Households	Smoking	Clustering of CVD RF (MetS)	Higher odds between ever smoking and MetS (OR 1.27, *p* = 0.037). Fewer current smokers were among those with MetS (OR 0.61, *p* < 0.001)
c Cross-sectional N = 686	Employees aged ≥ 35 year All: 45.9 ± 6.5	Kuala Lumpur	University	Physical activity, total sitting time	Clustering of CVD RF (MetS)	Highest sitting quartiles > 9.3 h/day and physically inactive were associated with increased odds for MetS (OR 5.38, 95% CI 2.75–10.52). Within the physically inactive stratum, those in the higher sitting quartiles of 7.6–9.29 h/day had an OR of 5.94 (95% CI 2.93–12.05) for MetS Moderate and high physical activity levels were associated with reduced odds (AOR 0.42, 95% CI 0.27–0.65; AOR 0.52; 95% CI 0.35–0.76) for MetS independent of sex Low activity levels in occupational (AOR 2.02, 95% CI 1.33–3.05), transportation (AOR 1.49, 95% CI 1.01–2.21) and household (AOR 1.96; 95% CI 1.33–2.91) domains were each associated with MetS
Mohd Noor et al. (2013) Cross-sectional N = 196	Adults between 40 and 70 year All: 55	Kelantan	Hospital	Physical activity, diet	10-year CVD risk (WHO/ISH risk)	3.7% of respondents have > 30% risk for CVD within the next 10 years. Diet and PA were not associated with the 10-year CVD risk
Chee et al., (2014a, 2014b) Cross-sectional N = 659	Government employees Female: 34.3 ± 8.9 Male: 35.0 ± 8.4	Putrajaya	Government agencies	Physical activity	Clustering of CVD RF (MetS)	Participants in pre-contemplation, contemplation and preparation stages of PA behaviour had higher odds of having MetS (OR 17.32, 95% CI 7.41–40.47; OR 12.74, 95% CI 6.19–26.25; OR 12.24, 95% CI 5.78–25.92) compared to the maintenance stage Participants in the action stage had a lower odd for MetS (OR 0.509, 95% CI 0.06–4.259)
Johari and Shahar (2014) Cross-sectional N = 343	Older adults ≥ 60 years All: 66.4 ± 5.9	Central Malaysia	Low-cost flats	Diet, physical activity	Clustering of CVD RF (MetS)	In men, high carbohydrate intake increases the odds for MetS (AOR 2.79, 95% CI 1.07–7.31) In women, physical inactivity (AOR 2.05, 95% CI 1.14–3.70) and good appetite (AOR 2.26, 95% CI 1.17–4.34) increases the odds for MetS
Rasiah et al. (2015) Cross-sectional N = 7276	Adults > 25 years	Nationwide	Community centers	Physical activity	Cumulative CVD RF	In the 35–49 age group, a moderate level of physical activity is associated with lower odds for a high number of CVD RF (AOR 0.12, 95% CI 0.02–0.53) In the > 65 years age group, a vigorous level of physical activity is associated with lower odds for a high number of CVD RF (AOR 0.58, 95% CI 0.24–0.78)
Shah et al. (2015) Retrospective study N = 21,862	Adults ≥ 18 years with CAD	Kuala Lumpur	Hospital	Smoking	Premature CAD	Smoking had a higher risk associated with premature CAD in 2007 (AOR 2.52, 95% CI 1.52–4.19) and 2012 (AOR 1.91, 95% CI 1.14–3.19)
Lim et al. (2016) Prospective cohort(REDISCOVER study) N = 10,805	Adults ≥ 30 years All: 52.6 ± 11.6	Nationwide	Households	Smoking	AF	Associations between AF and current smoking (AOR 1.18, 95% CI 0.73–1.91) and previous smoking (AOR 1.48, 95% CI 0.94–2.33) statuses were not significant
Borhanuddin et al. (2018) Prospective cohort(The Malaysian Cohort (TMC) Study) N = 53,122	Adults between 35 and 65 years Female: 48.3 ± 7.6 Male: 49.7 ± 7.9	Nationwide	Malaysia	Physical activity	10-year CVD risk-based (FRS-lipid or FRS-BMI)	Males with high PA (adj geometric mean estimate = 12.2%, 95% CI 12.0,1 2.4%) and moderate PA (12.3%, 95% CI 12.1, 12.5) have significantly lower CVD risk (FRS-lipid) compared to low PA. Similarly, moderate and high levels of PA remained protective against CVD risk using FRS-BMI Such effects were not seen in females
Ching et al. (2018) Cross-sectional N = 273	Adult (> 18 years) vegetarians All: 47.5 ± 13.1	Klang Valley	Community centres	Diet	Clustering of CVD RF (MetS)	Years of practising vegetarianism were associated with MetS when adjusted for sex (AOR 1.03, 95% CI 1.00–1.07), but the association was diminished when adjusted for sex and age
Lim et al. (2018) Cross-sectional N = 494	Employees between 40 and 65 years All: 46.8 ± 5.3	Klang Valley	Manufacturing factories	Working hours	Clustering of CVD RF (MetS)	Night workers were more likely to be associated with MetS than non-night-shift workers (OR 1.90, 95% CI 1.25–2.88) The odds for MetS were highest among day-evening-night shift workers (OR 3.27, 95% CI 1.78–5.99), followed by day–night shift workers (OR 2.06, 95% CI 1.19–3.56) and permanent night-shift workers (OR 1.85, 95% CI 0.41–8.34) compared with permanent daytime workers No association between the duration of shift work and MetS
Rashid et al. (2019) Case–control N = 142	Women with IHD between 30 and 65 years Case: 52.6 ± 8.6 Control: 52.3 ± 9.0	Terengganu	Hospital	Smoking, diet (fat intake), physical activity	IHD	The odds of IHD for passive smoking were even higher with women who were passive smokers at 3 times higher odds of getting IHD (AOR 2.99, 95% CI 1.81–4.94) than those who were not passive smokers Other RFs were not significant
Balasubramanian et al. (2020) Cross-sectional (Malaysia Lipid Study) N = 562	Adults 20–65 years All: 38.1 ± 11.4	Kuala Lumpur, Selangor	Households	Diet	Clustering of CVD RF(MetS)	Diet high in sugar-sweetened beverages was associated with increased risk for MetS (OR 2.78, 95% CI 1.49–5.22)
Iqbal et al. (2020) Cross-sectional N = 481	Adults > 18 years	Johor	Community	Diet, physical activity, alcohol	Clustering of CVD RF(MetS)	There are higher odds for MetS for quick finishing of meals among the Malays (AOR 2.17, 95% CI 1.02–4.60) and low PA among the Chinese (AOR 4.76, 95% CI 1.49–15.26). There is no significant association between alcohol intake and MetS
Juhari et al. (2020) Cross-sectional N = 180	Female nurses > 40 years All: 49.0 ± 4.5	Kelantan	Hospital	Physical activity, diet, working hours	10-year CVD risk (FRS)	Increased odds for moderate-high risk for CVD with physical inactivity (AOR 3.52, 95% CI 1.68–7.38), unhealthy diet (low FV intake) (AOR 12.44, 95% CI 2.80–55.20) and shift work (AOR 9.40, 95% CI 4.38–20.21)
Kuan et al. (2020) Cross-sectional N = 494	Healthcare employees between 19 and 59 years All: 32.4 ± 8.4	Sarawak	Hospital	Simple Lifestyle Indicator Questionnaire (SLIQ)	Cardiac risk	Cardiac risk was not significantly associated with a healthier lifestyle (OR 0.7, 95% CI 0.0–11.6)
Shariff et al. (2020) Cross-sectional (NHMS 2015) N = 3375	Adults > 60 years All: 68.1	Nationwide	Households	Physical activity	10-year CVD risk (FRS)	Physical inactivity is associated with lower moderate and high 10-year CVD risk (OR 0.72, 95% CI 0.55–0.95)
Chan et al. (2021) Cross-sectional (NHMS 2018) N = 7117	Adults > 55 years All: 62.2 ± 8.8	Nationwide	Households	Diet (FV intake), physical activity	Clustering of CVD RF	No significant association between FV intake and the clustering of CVD risk factors Odds of having ≥ 2 (AOR 1.27, 95% CI 1.02–1.59) or ≥ 3 (AOR 1.38, 95% CI 1.07–1.77) modifiable CVD risk factors were higher among older adults who were physically inactive
Manaf et al. (2021) Cross-sectional N = 538	Employees between 26 and 60 years All: 43.4 ± 7.7	Selangor	University	Physical activity, alcohol	Clustering of CVD RF(MetS)	Physical activity with moderate intensity was associated with MetS (AOR 1.8, 95% CI 1.1–1.3). The association diminished after stratification according to sex No association between alcohol and MetS
Abu Bakar et al. (2022) Wan Musa et al. (2022) (MyDiet-CHD) Cross-sectional N = 365	Adults between 30 and 64 years All: 51.6 ± 9.1	Nationwide	Medical clinics	Diet, smoking	Recent CHD diagnosis	Dietary patterns characterised by “high saturated fatty acid, high dietary energy density (DED), high sodium” were associated with CHD (AOR 1.32, 95% CI 1.03–1.69) Total fat (AOR 1.04, 95% CI 1.02–1.05) and full cream dairy product (AOR 1.004, 95% CI 1.001–1.008) intakes, and smoking (AOR 4.69, 95% CI 2.40–9.18) were associated with increased odds for CHD
Ang et al. (2022) Cross-sectional (SEACO) N = 11,897	Adults > 35 years	Johor	Households	Diet, physical activity	10-year CVD risk (FRS)	Lower risk for CVD with lesser frequency of meals taken outside: 11 meals versus 0 meals: b = − 0.342 (95% CI − 0.56 to − 0.12) and 11 meals versus 1–5 meals: b = − 0.572 (95% CI 0.78 to − 0.367) Physical activity not associated with CVD risk
Hasbullah et al. (2022) Cross-sectional (MyNutritype cohort) N = 157	Adult women with a history of GDM All: 34.8 ± 5.6	Selangor	Health clinics	Diet	Clustering of CVD RF(MetS)	Western and prudent dietary patterns were not found to be associated with MetS in women with a history of GDM
Lee et al. (2022) Cross-sectional (CLUSTer cohort) N = 1151	Teachers All: 42.3 ± 9.1	Malacca	Schools	Diet, smoking, sleep, physical activity, alcohol	Clustering of CVD RF(MetS)	Usage of saturated fat (OR 1.34, 95% CI 1.01 = 1.79) and lesser sleeping duration (OR 1.46, 95% CI 1.13–1.89) were associated with increased odds for MetS Smoking, physical activity, sitting time, fruit and vegetable intake, alcohol consumption were not associated with MetS

*OR* odds ratio, *AOR* adjusted odds ratio, *95% CI* 95% confidence interval, *CVD RF* cardiovascular disease risk factors, *MetS* metabolic syndrome, *CAD* coronary artery disease, *CHD* coronary heart disease, *IHD* ischemic heart disease, *AF* atrial fibrillation

**Table 2:** Studies reported on interventions for cardiovascular diseases among Malaysians (n = 8)

Study, Study design, Sample size	Target population Mean age ± SD (years)	Location	Intervention setting	Intervention focus	Duration of intervention	Main outcomes
Shahar et al. (2013) Quasi-experimental N = 42	Older adults with MetS IG: 65.3 ± 3.5 CG: 67.8 ± 4.7	Selangor	Rural community	Nutrition education	6 months	Women in IG showed a reduction in waist circumference compared to CG Men in IG maintained total cholesterol levels compared to CG
Chee et al. (2014b) RCT N = 120	Adults with MetS 18–59 years	Putrajaya	Facebook	Physical activity	6 months	IG showed a greater number of steps/day increase than CG. The number of steps were correlated with significant changes in all indicators of MetS At post-intervention, IG experienced a greater reduction in MetS (− 94%) than CG (− 22%), compared to baseline
Yaacob et al. (2016) Randomized trial N = 89	Adults with BMI ≥ 23.0 kg/m^2^ Dumbbell group: 37.9 ± 6.9 Ankle-wrist weight group: 40.1 ± 8.6	Kelantan	Community	Physical activity	2 years	The dumbbell group experienced reductions in BMI at week 6 Improvements in WC, WHR, body fat % and skeletal muscle % in both groups from baseline at week 6, month 3, and month 6
Hassan et al. (2017) RCT N = 64	Perimenopausal women IG: 50.6 ± 3.5 CG: 49.8 ± 2.7	Kelantan	Unknown	Multifaceted	2 years	Reduction in mean SBP in the intervention group (d = − 8.78 mmHg) at post-intervention, compared to baseline
Chee et al. (2017) RCT N = 155	Government employees IG: 36.2 ± 9.8 CG: 35.2 ± 9.2	Putrajaya	Worksite	Physical activity	6 months	Strong negative correlation between change in the number of steps per day and body weight, BMI, body fat percentage, fat mass, waist circumference, hip circumference, WHR, SBP, DBP, TC, LDL-C Moderate negative correlations were found with TG and FPG Moderate correlation found with HDL-C
Omar et al. (2018) Quasi-experimental N = 179	Housewives living in low-cost flats 18–59 years	Klang Valley	Community	Physical activity	1 year	IG showed a significant reduction in TG, albeit with a small effect size (0.08) No differences in terms of glucose and lipid profile
Mahadzir et al. (2020) Pre-post trial N = 48	Adults with metabolic syndrome Median = 46 years (IQR = 11)	Johor	Community	Multifaceted	6 months	Reduction in SBP (d = − 3.54%), FBG (d = − 11.98%), BMI (d = − 1.63%) and TG (d = − 24.22%) between baseline and post-intervention, but not between post-intervention and post-6 months follow-up A smaller decrease in WC at post-intervention (d = − 0.71%) An increase was observed in HDL-C at the end of the intervention (d = + 25.89%)
Omar et al. (2021) RCT N = 70	Young adult males at risk for CVD IG: 26.2 ± 6.9 CG: 26.6 ± 7.4	Selangor	Institute of vocational skills for youth	Physical activity	3 months	Significant improvement in anthropometric variables (weight, BMI, waist circumference) and lipid (TG, HDL) (time and group effect)

*d* difference, *RCT* randomized-controlled trial, *MetS* metabolic syndrome, *IG* intervention group, *CG* control group, *BMI* body mass index, *WC* waist circumference, *WHR* waist-hip ratio, *SBP* systolic blood pressure, *DBP* diastolic blood pressure, *TC* total cholesterol, *TG* triglyceride, *FPG* fasting plasma glucose, *LDL-C* low density lipoprotein cholesterol, *HDL-C* high density lipoprotein cholestero

**Table 3:** Characteristics of the included studies (N = 31)

	n	References
*Risk factors for cardiovascular diseases (n* = *23)*		
Study design		
Cross-sectional	19	Yeow et al. (2012), Chu and Moy (2013a, 2013b, 2014), Mohd Noor et al. (2013), Chee et al. (2014a), Johari and Shahar (2014), Ching et al. (2018), Lim et al. (2018), Balasubramanian et al. (2020), Iqbal et al. (2020), Juhari et al. (2020), Kuan et al. (2020), Shariff et al. (2020), Chan et al. (2021), Manaf et al. (2021), Abu Bakar et al. (2022), Ang et al. (2022), Wan Musa et al. (2022), Hasbullah et al. (2022), Lee et al. (2022), Rasiah et al. (2015)
Prospective cohort	2	Lim et al. (2016), Borhanuddin et al. (2018)
Retrospective cohort	1	Shah et al. (2015)
Case–control	1	Rashid et al. (2019)
Location		
Nationwide	7	Yeow et al. (2012), Rasiah et al. (2015), Lim et al. (2016), Borhanuddin et al. (2018), Shariff et al. (2020), Chan et al. (2021), Abu Bakar et al. (2022), Wan Musa et al., (2022
Selected states	16	Chu and Moy (2013a, 2013b, 2014), Mohd Noor et al. (2013), Chee et al. (2014a), Johari and Shahar (2014), Shah et al. (2015), Ching et al. (2018), Lim et al. (2018), Rashid et al. (2019), Balasubramanian et al. (2020), Iqbal et al. (2020), Juhari et al. (2020), Kuan et al. (2020), Manaf et al. (2021), Ang et al. (2022), Hasbullah et al. (2022), Lee et al. (2022)
Study setting		
Households/community	11	Yeow et al. (2012), Johari and Shahar (2014), Rasiah et al. (2015), Borhanuddin et al. (2018), Ching et al. (2018), Balasubramanian et al. (2020), Iqbal et al. (2020), Shariff et al. (2020), Chan et al. (2021), Lim et al. (2016), Ang et al. (2022)
Hospital/clinic	7	Mohd Noor et al. (2013), Juhari et al. (2020), Kuan et al. (2020), Abu Bakar et al. (2022), Wan Musa et al. (2022), Hasbullah et al. (2022), Shah et al. (2015), Rashid et al. (2019)
Worksite	5	Chee et al. (2014a), Chu and Moy (2013a, 2013b, 2014), Lim et al. (2018), Manaf et al. (2021), Lee et al. (2022)
Lifestyle risk factors		
Physical activity	13	Chu and Moy (2013a, 2013b, 2014), Mohd Noor et al. (2013), Chee et al. (2014a), Johari and Shahar (2014), Rasiah et al. (2015), Borhanuddin et al. (2018), Iqbal et al. (2020), Juhari et al. (2020), Shariff et al. (2020), Chan et al. (2021), Manaf et al. (2021), Rashid et al. (2019), Ang et al. (2022)
Diet	12	Mohd Noor et al. (2013), Johari and Shahar (2014), Ching et al. (2018), Balasubramanian et al. (2020), Iqbal et al. (2020), Juhari et al. (2020), Chan et al. (2021), Abu Bakar et al. (2022), Wan Musa et al. (2022), Hasbullah et al. (2022), Lee et al. (2022), Rashid et al. (2019), Ang et al. (2022)
Smoking	6	Yeow et al. (2012), Shah et al. (2015), Lee et al. (2022), Lim et al. (2016), Rashid et al. (2019), Wan Musa et al. (2022)
Alcohol		Iqbal et al. (2020), Lee et al. (2022)
Sleep/working hours	3	Lim et al. (2018), Juhari et al. (2020), Lee et al. (2022)
Overall lifestyle	1	Kuan et al. (2020)
*Intervention studies (n* = *8)*		
Study design		
Quasi-experimental	2	Shahar et al. (2013), Omar et al. (2018)
Randomised trials	5	Chee et al. (2014b), Yaaccob et al. (2016), Hassan et al. (2017), Chee et al. (2017), Omar et al. (2021)
Pre-post trial	1	Mahadzir et al. (2020)
Location		
Selected states	8	Shahar et al. (2013), Chee et al. (2014b), Yaaccob et al. (2016), Hassan et al. (2017), Chee et al. (2017), Omar et al. (2018), Mahadzir et al. (2020), Omar et al. (2021)
Study setting		
Community	4	Shahar et al. (2013), Yaaccob et al. (2016), Omar et al. (2018), Mahadzir et al. (2020)
Online	1	Chee et al. (2014b)
Workplace	1	Chee et al. (2017)
Institute	1	Omar et al. (2021)
Unknown	1	Hassan et al. (2017)
Intervention focus		
Physical activity	5	Yaaccob et al. (2016), Chee et al. (2014b), Chee et al. (2017), Omar et al. (2018), Omar et al. (2021)
Multifaceted lifestyle	2	Hassan et al. (2017), Mahadzir et al. (2020)
Nutrition	1	Shahar et al. (2013)

On the other hand, a randomised trial was the most common study design that investigated the impact of lifestyle interventions on CVDs (n = 5). None of these interventions were conducted nationwide, though four were focused on the communities. Physical activity was the most common intervention focus (n = 5). The duration of the intervention varied considerably from less than 2 weeks to 2 years.

### Lifestyle-Related Risk Factors for Cardiovascular Diseases

#### Physical Activity

Table 4 summarises associations reported between lifestyle risk factors and CVD. Physical inactivity has been consistently associated with increased odds of CVD risk, with a significant positive association found in eight studies (Borhanuddin et al., 2018; Chan et al., 2021; Chee et al., 2014a; Chu & Moy, 2013a, 2013b, 2014; Iqbal et al., 2020; Johari & Shahar, 2014; Juhari et al., 2020; Rasiah et al., 2015).

**Table 4:** Association between lifestyle factors and cardiovascular diseases (n = 21)

	Lifestyle risk factors					
	Physical inactivity	Diet	Smoking	Working hours	Alcohol	Healthy lifestyle
MetS						
10-year CVD risk						
Clustering of CVD RF						
Cardiac risk						
CVD diagnosis						
References	Chu and Moy (2013a, 2013b, 2014), Mohd Noor et al. (2013), Chee et al. (2014a), Johari and Shahar (2014), Borhanuddin et al. (2018), Iqbal et al. (2020), Juhari et al. (2020), Shariff et al. (2020), Chan et al. (2021), Manaf et al. (2021), Ang et al. (2022) and Rasiah et al. (2015)	Mohd Noor et al. (2013), Johari and Shahar (2014), Ching et al. (2018), Rashid et al. (2019), Juhari et al. (2020), Balasubramanian et al. (2020), Iqbal et al. (2020), Chan et al. (2021), Lee et al. (2022), Abu Bakar et al. (2022), Ang et al. (2022), Wan Musa et al. (2022) and Hasbullah et al. (2022)	Yeow et al. (2012), Shah et al. (2015), Lim et al. (2016), Rashid et al. (2019), Lee et al. (2022) and Wan Musa et al. (2022)	Lim et al. (2018), Juhari et al. (2020)	Iqbal et al. (2020), Manaf et al. (2021) and Lee et al. (2022)	Kuan et al. (2020)

*MetS* metabolic syndrome, *CVD RF* cardiovascular disease risk factors

: Significant reduced risk/odds

: Significant increased risk/odds

: No significant association

Specifically, four studies reported a positive association between physical inactivity and metabolic syndrome (MetS). Among working adults, moderate and high physical activity levels were associated with almost 35–60% reduced odds for MetS independent of sex (Chu & Moy, 2013b). Chu and Moy (2013a) reported that those who sat more than 6 h daily had more than three times higher odds for metabolic risk factors. Low activity levels in occupational, transportation and household domains were each positively associated with MetS (Chu & Moy, 2014). Government employees in the pre-contemplation, contemplation and preparation stages of physical activity behaviour had higher odds of having MetS compared to the maintenance stage (Chee et al., 2014a). In a study conducted in the community, Iqbal and colleagues (2020) reported that low physical activity had nearly five times higher odds for MetS among the Chinese population, which was the largest effect seen among these studies. Inactive elderly women have been reported to have twice the odds of MetS compared to physically active ones (Johari & Shahar, 2014).

Two studies reported a positive association between inactivity and 10-year CVD risk (Borhanuddin et al., 2018; Juhari et al., 2020), while one reported similar findings with clustering of CVD risk factors (Chan et al., 2021). Male participants of The Malaysian Cohort Study with high and moderate physical activity have significantly lower 10-year CVD risk than males with low physical activity (Borhanuddin et al., 2018). An increased odds for moderate 10-year CVD risk was seen among female nurses in Kelantan with physical inactivity (Juhari et al., 2020). Unusually, Shariff et al. (2020) reported that physical inactivity among older adults from the National Health Morbidity Survey (NHMS) 2015 is associated with a 30% lower moderate and high 10-year CVD risk. However, Chan and colleagues (2021) utilised updated NHMS 2018 data to show a significant 27–38% increase in the odds for modifiable CVD risk factors in older adults. A potential explanation for the discrepancy observed by Shariff et al. (2020) could result from the limitations of the Framingham Risk Score (FRS) in accurately estimating coronary heart disease (CHD) risk among older adults, especially women. This may arise as the FRS tends to underestimate risk due to age-related shifts in the associations between traditional risk factors and CHD. For instance, total and LDL cholesterol, though being a strong cardiovascular risk factor in middle-aged adults, exhibit weaker associations with CHD risk in older adults.

Whereas, Rasiah et al. (2015) reported possible differences in the association between physical activity and cumulative CVD risk based on age. In their nationwide study, the researchers discovered that in the lower age group (35–49 years), a moderate level of physical activity appeared more protective, while a vigorous level of physical activity was associated with lower odds for CVD risk in those above 65 years.

#### Diet

From 12 studies that investigated diet-CVD risk association, seven studies (Abu Bakar et al., 2022; Ang et al., 2022; Balasubramanian et al., 2020; Iqbal et al., 2020; Johari & Shahar, 2014; Juhari et al., 2020; Lee et al., 2022; Wan Musa et al., 2022) reported a significant association between different aspects of diet and increased odds of CVD-related risk in the Malaysian community (Table 4).

Evidence suggested an increased odds for MetS for higher consumption of carbohydrates in men (Johari & Shahar, 2014), good appetite in women (Johari & Shahar, 2014), sugar-sweetened beverages (SSB) (Balasubramanian et al., 2020), quick finishing of meals among Malays (Iqbal et al., 2020) and usage of saturated fat in cooking (Lee et al., 2022). In addition, findings from the nationwide MyDiet-CHD study showed diets high in saturated fatty acids, energy density, and sodium were associated with recent CHD diagnosis (Abu Bakar et al., 2022). Juhari and colleagues (2020) reported that unhealthy diets, particularly with low vegetable and fruit intake, increased the odds of a moderate-high 10-year risk for CVD. The same study also showed increased odds of CHD with higher total and full-cream dairy product intakes (Wan Musa et al., 2022). In another community observatory, data collected from 11,897 respondents revealed a lower 10-year risk for CVD associated with a reduced frequency of non-home-cooked meals taken away from home per week (Ang et al., 2022).

However, there were some contradictory findings reported by the remaining studies. Overall dietary intake and patterns were not associated with Mets (Ching et al., 2018; Hasbullah et al., 2022), 10-year CVD risk (Mohd Noor et al., 2013), IHD diagnosis (Rashid et al., 2019) and clustering of CVD risk factors in older adults (Chan et al., 2021). These inconsistencies may be attributed to various dietary assessment methods and the targeting of different study populations. For example, in the study by Ching et al. (2018), a predominantly vegetarian participant base may have influenced the findings. Additionally, differences in the methodologies employed for dietary assessment, such as the Short Fat Questionnaire used in the study by Rashid et al. (2019) compared to the more extensive 165-item semi-quantitative food frequency questionnaire (FFQ) utilised by Hasbullah et al. (2022), could also account for discrepancies in results.

#### Smoking

Relatively, fewer studies explored the relationship between smoking and CVD-related risk (Table 3). Of six studies that investigated the smoking-CVD link (Lee et al., 2022; Lim et al., 2016; Rashid et al., 2019; Shah et al., 2015; Wan Musa et al., 2022; Yeow et al., 2012), four reported a significant positive association (Rashid et al., 2019; Shah et al., 2015; Wan Musa et al., 2022; Yeow et al., 2012).

Most of the studies that explored this relationship were focused on recent CVD diagnoses. Smoking was associated with as high as 2.5 times higher odds of premature coronary artery disease (CAD) (Shah et al., 2015) and almost five times higher odds of CHD diagnosis (Wan Musa et al., 2022). Women exposed to passive smoking had three times increased odds for IHD (Rashid et al., 2019). In addition to this, a nationwide study among 4341 participants found a smoking history associated with 27% higher odds of MetS (Yeow et al., 2012), though this association was not replicated in the CLUSTer cohort a decade later (Lee et al., 2022). Similarly, Lim and colleagues (2016) did not find a significant association between smoking and AF. The authors postulated that this could be attributed to the higher proportion of non-smokers within the study population.

#### Other Lifestyle Factors

Working hours, alcohol intake, and overall lifestyle were other lifestyle-related factors explored in terms of their relationship with CVD risk (Table 4). The odds for MetS were highest among day-evening-night shift workers of manufacturing factories (Lim et al., 2018). The day-evening-night shift pattern described in the study entails workers transitioning between day, evening, and night shifts. This arrangement often results in shorter sleep duration, increased sleep disturbances, and daytime dysfunction (Lim et al., 2018). In another study, nurses who practised shift work had more than nine times higher increased odds for moderate-high risk CVD compared to nurses who do not work on a shift basis (Juhari et al., 2020). No significant association was found between overall lifestyle and cardiac risk (Kuan et al., 2020), as well as alcohol intake and MetS (Iqbal et al., 2020; Lee et al., 2022; Manaf et al., 2021).

### Primary Prevention of Cardiovascular Diseases

Eight studies assessed the intervention effects of primary prevention on CVD (Table 2). Five of these interventions focused solely on physical activity (n = 5), followed by a multifaceted lifestyle approach (n = 2) and nutrition (n = 1) (Table 3).

A physical activity-focused randomised controlled trial (RCT) (Chee et al., 2014b) conducted via social media among adults with MetS showed significant improvement in cardiometabolic measures, including anthropometry, blood pressure, lipid profile, and blood glucose level. Another trial with a similar theme and duration (6 months) but conducted at a worksite, showed a significant effect on blood glucose (Chee et al., 2017). These findings were echoed by Omar et al. (2021), who investigated the effect of a 12-week pedometer-based walking intervention for primary prevention of CVD among young males at risk for CVD. The findings demonstrated significant improvements in body weight, WC, BMI, and lipid profile. Additionally, the quasi-experiment also showed a notable improvement in other secondary outcomes such as C-reactive protein (CRP), Interleukin-6 (IL-6), and Tumor Necrosis Factor ɑ (TNFɑ) levels (time and group effect). Longer duration of physical activity intervention resulted in a significant effect only on triglyceride levels, but not on other markers (Omar et al., 2018). Interestingly, Yaacob and colleagues (2016) investigated the effect of using different tools (ankle-wrist weight and dumbbells) to elucidate the impact of physical activity on anthropometry. Both groups experienced improved WC, waist-hip ratio (WHR), body fat and skeletal muscle, while an additional benefit in BMI was seen only in the dumbbell group.

A multifaceted approach included health education programmes, counselling, focus groups, self-monitoring booklets, and exercise (Hassan et al., 2017; Mahadzir et al., 2020) (Table 2). Perimenopausal women who underwent a 2-year multifaceted intervention showed significant improvement in systolic blood pressure (SBP) and diastolic blood pressure (DBP) (Hassan et al., 2017), while another group-based feasibility study that took on a multifaceted approach showed significant improvement in SBP, fasting blood glucose (FBG), BMI, triglycerides (TG), WC and HDL cholesterol levels among adults with MetS (Mahadzir et al., 2020). On the other hand, Shahar et al. (2013) conducted a quasi-experimental study adopting a nutrition education-only method to yield significant improvements in body weight and BMI among male participants while significantly affecting WC in female participants and body weight post-intervention.

## Discussion

Our review showed most consistent evidence for CVD risk for physical inactivity (Borhanuddin et al., 2018; Chan et al., 2021; Chee et al., 2014a; Chu & Moy, 2013a, 2013b, 2014; Iqbal et al., 2020; Johari & Shahar, 2014; Juhari et al., 2020; Manaf et al., 2021; Mohd Noor et al., 2013; Shariff et al., 2020), and smoking (Rashid et al., 2019; Shah et al., 2015; Wan Musa et al., 2022; Yeow et al., 2012). Unhealthy diets were characterised by diverse factors including high carbohydrate intake (Johari & Shahar, 2014), SSB (Balasubramanian et al., 2020), quick finishing of meals (Iqbal et al., 2020), low fruit and vegetable intakes (Juhari et al., 2020), total fat (Wan Musa et al., 2022), full cream dairy products (Wan Musa et al., 2022), saturated fat use (Lee et al., 2022) and overall poor dietary patterns (Abu Bakar et al., 2022), which increases the odds for CVD-related risk. Besides, shift work was found to be associated with CVD in two studies (Juhari et al., 2020; Lim et al., 2018), while lack of optimal health screening was found to be associated with a clustering of lifestyle risks, namely smoking, physical inactivity, and unhealthy diet (Kuan et al., 2020).

Several previously published systematic reviews support our findings. In their meta-analysis, Pan and colleagues (2015) found that smoking increases the risk of total mortality and cardiovascular events in people with diabetes, with men who smoke one cigarette daily having a higher risk of CHD than expected. The effect also appears to be sex-specific. It’s worth noting that the majority of studies included in their analysis were conducted in European countries, with additional research from countries such as the United States, Australia, New Zealand, and China. One meta-analysis by Hackshaw et al. (2018) emphasised that considering CHD as the outcome, men who smoked one cigarette per day had 46% of the excess relative risk for smoking 20 cigarettes per day compared to women who had 31% of the excess risk. Their analysis included a broader study population, and only a few were conducted in Asia–Pacific countries such as Japan, China, South Korea, Hong Kong, and Taiwan.

A systematic review and meta-analysis conducted by Paudel et al. (2019) reported that inactive South Asian adults had a 34% increased risk of exhibiting poor cardio-metabolic outcomes compared to those with moderate or higher physical activity levels. Physical inactivity is also associated with an increased risk of diabetes, higher BMI, waist circumference, BP, and glucose levels in a review conducted in the South Asian population (Ahmad et al., 2017).

The association between fruits and vegetables and CVD risk reported in our review can be supported by a previous systematic review by Aune et al. (2017), which found negative correlations between fruits and vegetables, and the risk of CHD, stroke, CVD, total cancer, and all-cause mortality, with specific varieties having inverse links with these diseases. Specifically, apples/pears, citrus fruits, cruciferous vegetables, green leafy vegetables, tomatoes, and fruit and vegetables high in beta-carotene and vitamin C were inversely linked with CHD, stroke, or CVD.

We also found shift workers to have increased odds of CVD compared to non-shift workers, with short sleep duration being a contributing factor. Similarly, Torquati et al. (2018) reported a 17% greater CVD risk among shift workers than among day workers in their systematic review. In addition, most of those who did not go for optimal screening for CVD have a clustering of lifestyle risk. According to Bloetzer et al. (2015), both population-based and high-risk prevention programs, such as screening, have helped to significantly reduce CVD in both sexes and across all age groups in the United States, Canada, and Switzerland.

Multiple interventions have been carried out to decrease the CVD risk with the most common interventions focusing on physical activity (Chee et al., 2014b, 2017; Omar et al., 2018, 2021; Yaccob et al., 2016) and multifaceted lifestyle changes (Hassan et al., 2017; Mahadzir et al., 2020), with main changes seen in anthropometry (Chee et al., 2017; Mahadzir et al., 2020; Omar et al., 2021; Yaacob et al., 2016), blood pressure (Hassan et al., 2017, Chee et al., 2017, Mahadzir et al., 2020 and blood biomarkers (Chee et al., 2017, Omar et al., 2018, Mahadzir et al., 2020, Omar et al., 2021). Other studies and reviews supported our findings. Other studies have identified Regular physical activity as an effective primary prevention strategy for CVD. For example, a longitudinal study by Ahmadi et al. (2022) found that increasing physical activity from any baseline level significantly reduced the risk of all-cause mortality and CVD mortality. Furthermore, a population-based prospective cohort study in China (Bennett et al., 2017) reported that an increase in daily physical activity equivalent to four metabolic equivalent task hours was linked to a reduced risk of CVD. In addition, Ostman et al. (2017) conducted a systematic review and meta-analysis investigating the effect of exercise training in patients with MetS and reported significant improvements in cardiovascular and metabolic outcome measures.

A meta-analysis of 12 multifaceted lifestyle interventions conducted in developed countries identified limited effects on major cardiovascular risk factors after 2 years of follow-up (Bergum et al., 2021). However, a modest effect on SBP and TC was noted, with smoking cessation leading to a greater reduction in smoking rates. In our review, educational interventions have also been explored as a means of modifying CVD risk factors. A systematic review found that BP was the most evident risk factor showing a significant decrease in favour of educational intervention (Rahmati Najarkolaei et al., 2015). Beneficial educational interventional programs were population-based, dietary educational sessions, and school-oriented weight loss programs for students. In addition, a systematic review primarily targeting the population in the USA has assessed self-management support interventions in chronically ill patients with low socioeconomic status (Van Hecke et al., 2017). While improvements were seen in the cardiac-specific quality of life and the incidence of death or hospitalisations, they were not statistically significant.

Finally, mobile health (mHealth) technology has been studied as a means of targeted cardiovascular care (Gandhi et al., 2017) and can be seen as a way forward in the primary prevention of CVD. Studies have shown significant adherence to medical, pharmacologic, and nonpharmacologic therapies, as well as a reduction in blood pressure and a decrease in transient ischemic attack/stroke recurrence in those with cerebrovascular disease. However, certain limitations should be considered, such as the exclusion of populations without smartphones and the reliance on text messaging for motivation and education. Furthermore, among the 27 studies included in this systematic review, only one study specifically focuses on Malaysia.

Our review offers novel insights into the multifaceted lifestyle interventions by shedding light on their relevance and implications for CVD prevention within the Malaysian context. However, we also recognise that there are still gaps that need to be addressed. These may include integrating multiple lifestyle interventions and limited studies specifically addressing multifaceted lifestyle interventions, which we believe merit further investigation to advance our understanding of their impact on CVD prevention. The utilisation of diverse research methodologies is important for enhancing the reliability and robustness of evidence in future studies. In this scoping review, we primarily utilised cross-sectional studies to examine lifestyle-related risk factors for CVDs in the Malaysian adult population, and fewer other study designs, such as cohorts (Lim et al., 2016, Borhanuddin et al., 2018; and Shah et al., 2015) and case–control (Rashid et al., 2019) have been used. As for the lifestyle-related intervention studies, the majority of the study designs were RCTs, while population-based quasi-experiments and pre-post trials were relatively rare (Shahar et al., 2013, Omar et al., 2018; Mahadzir et al., 2020). While cross-sectional studies offer valuable insights into the prevalence and associations of lifestyle-related risk factors for CVDs, they inherently possess limitations, such as the inability to establish causality or temporality. By incorporating alternative study designs such as cohorts and case–control studies, future research can provide a more comprehensive understanding of the temporal relationships between risk factors and CVD outcomes.

Furthermore, while RCTs are considered the gold standard for evaluating the efficacy of interventions, there is a limitation to the generalisability of the findings. Population-based quasi-experiments allow for evaluating natural experiments or policy interventions, providing real-world evidence of intervention effectiveness in the community. Pre-post trials, on the other hand, offer insights into the effectiveness of interventions within specific populations or settings, facilitating the identification of practical strategies for CVD prevention and management. Hence, future research should diversify its approach by incorporating population-based quasi-experiments and pre-post trials alongside RCTs. By adopting a multi-methodological approach, future studies can address the inherent limitations of individual study designs while leveraging the strengths of diverse methodologies to generate more reliable and comprehensive evidence.

Additionally, we identified certain aspects which have remained underexplored in Malaysia. For example, alcohol consumption has not been studied much within the Malaysian context despite its significance in other populations. Zhang et al. (2021) reported that 350 g/week of alcohol consumption was associated with a higher risk of stroke in the Kailuan community in China. The limited study of alcohol intake in Malaysia can be attributed to religious reasons, as the country’s population is predominantly Muslim, with the majority of participants with alcohol consumption being predominantly limited to non-Muslim communities. This unique sociocultural context presents a barrier to comprehensive research on alcohol’s effects and patterns within the Malaysian population.

### Strengths and Limitations

Our scoping review has several strengths. We thoroughly analyzed various databases to determine the lifestyle risk factors and interventions reportedly correlating with cardiovascular-related health outcomes. A robust methodology was utilised to evaluate the quality of each study. Two reviewers were involved during the screening and inclusion process to minimise selection bias, with a third reviewer confirming the final list of articles by resolving conflicts and overseeing the process. The strengths of our review are the focus on lifestyle risk factors and interventions, the inclusion of a wide range of study designs, and cohorts that cover various geographical locations in Malaysia that are representative of LMICs. An effort was made to meticulously incorporate relevant articles with no specific impositions on the types of interventions and definitions of CVD. Additionally, we only included human interventional studies to ensure a high quality of evidence for future implications in clinical practice.

Some limitations in our review need to be considered. Firstly, it is difficult to compare research data when there are several ways of measuring the physical activity levels among the participants. In contrast to the studies that assessed physical activity levels with the International Physical Activity Questionnaire (IPAQ) (Borhanuddin et al., 2018; Chu & Moy, 2013a; Iqbal et al., 2020; Rashid et al., 2019) and Global Physical Activity Questionnaire (GPAQ) (Chan et al., 2021; Juhari et al., 2020), other studies assessed physical activity according to their own definition. Hence, with different definitions, participants can be regarded as physically active in one study and inactive in another. Therefore, a standardised definition for assessing physical activity levels is needed to establish accuracy. Additionally, some studies used self-reported data, which is highly susceptible to recall, interviewer, and reporting bias.

## Conclusions

The findings suggest improving lifestyle-related risk assessments and interventions for the prevention of cardiovascular diseases in this population. Although some positive outcomes have been noted, it remains unclear whether these findings directly translate into improved interventions for preventing CVD. Therefore, additional research is necessary to establish a definitive correlation between these outcomes and their potential impact on efforts to prevent CVD. Nevertheless, intervention using the multifaceted lifestyle approach can improve CVD-related outcomes.

A key implication of this review is the importance of addressing the imbalanced proportion of the studies on different lifestyle risk factors and interventions. For instance, few studies have reported on the association between stress, diet, and working hours therefore, there is an evident information gap in the current studies. It is recommended that more nationwide population-based interventions be conducted focusing on lifestyle interventions and their impact on cardiovascular disease prevention to enhance the validity of the results.

### Supplementary Information

Below is the link to the electronic supplementary material.

Supplementary file1 (DOCX 16 KB)

## References

[CR1] Abdo Ahmed, A. A., Mohammed Al-Shami, A., Jamshed, S., Zawiah, M., Elnaem, M. H., & Mohamed Ibrahim, M. I. (2020). Awareness of the risk factors for heart attack among the general public in Pahang, Malaysia: A cross-sectional study. *Risk Management Healthcare Policy,**13*, 3089–3102. 10.2147/RMHP.S281285 33380849 10.2147/RMHP.S281285 PMC7767710

[CR2] Abu Bakar, N. A. F., Ahmad, A., Wan Musa, W. Z., Shahril, M. R., Wan-Arfah, N., Abdul Majid, H., Piernas, C., Ramli, A. W., & Naing, N. N. (2022). Association between a dietary pattern high in saturated fatty acids, dietary energy density, and sodium with coronary heart disease. *Scientific Reports,**12*(1), 13049. 10.1038/s41598-022-17388-5 35906378 10.1038/s41598-022-17388-5 PMC9336144

[CR3] Ahmad, S., Shanmugasegaram, S., Walker, K. L., & Prince, S. A. (2017). Examining sedentary time as a risk factor for cardiometabolic diseases and their markers in South Asian adults: A systematic review. *International Journal of Public Health,**62*(4), 503–515. 10.1007/s00038-017-0947-8 28299392 10.1007/s00038-017-0947-8

[CR4] Ahmadi, M. N., Lee, I. M., Hamer, M., Del Pozo Cruz, B., Chen, L. J., Eroglu, E., Lai, Y. J., Ku, P. W., & Stamatakis, E. (2022). Changes in physical activity and adiposity with all-cause, cardiovascular disease, and cancer mortality. *International Journal of Obesity,**46*(10), 1849–1858. 10.1038/s41366-022-01195-z 35915134 10.1038/s41366-022-01195-z PMC9492547

[CR5] Ang, C. W., Ismail, R., Kassim, Z., Mohd Ghazali, A. N., Reidpath, D., & Su, T. T. (2022). Frequent eating out and 10-year cardiovascular disease risk: Evidence from a community observatory in Malaysia. *BioMed Research International,**2022*, 2748382. 10.1155/2022/2748382 35295957 10.1155/2022/2748382 PMC8920648

[CR6] Aune, D., Giovannucci, E., Boffetta, P., Fadnes, L. T., Keum, N., Norat, T., Greenwood, D. C., Riboli, E., Vatten, L. J., & Tonstad, S. (2017). Fruit and vegetable intake and the risk of cardiovascular disease, total cancer and all-cause mortality—A systematic review and dose–response meta-analysis of prospective studies. *International Journal of Epidemiology,**46*(3), 1029–1056. 10.1093/ije/dyw319 28338764 10.1093/ije/dyw319 PMC5837313

[CR7] Balasubramanian, G. V., Chuah, K. A., Khor, B. H., Sualeheen, A., Yeak, Z. W., Chinna, K., Sundram, K., & Karupaiah, T. (2020). Associations of eating mode defined by dietary patterns with cardiometabolic risk factors in the Malaysia Lipid Study population. *Nutrients,**12*(7), 2080. 10.3390/nu12072080 32674327 10.3390/nu12072080 PMC7400910

[CR8] Bennett, D. A., Du, H., Clarke, R., Guo, Y., Yang, L., Bian, Z., Chen, Y., Millwood, I., Yu, C., He, P., Zheng, X., Collins, R., Chen, J., Peto, R., Li, L., Chen, Z., China Kadoorie Biobank Study Collaborative Group. (2017). Association of physical activity with risk of major cardiovascular diseases in Chinese men and women. *JAMA Cardiology,**2*(12), 1349–1358. 10.1001/jamacardio.2017.4069 29117341 10.1001/jamacardio.2017.4069 PMC5814992

[CR9] Bergum, H., Sandven, I., & Klemsdal, T. O. (2021). Long-term effects (> 24 months) of multiple lifestyle intervention on major cardiovascular risk factors among high-risk subjects: A meta-analysis. *BMC Cardiovascular Disorders,**21*(1), 181. 10.1186/s12872-021-01989-5 33858345 10.1186/s12872-021-01989-5 PMC8048075

[CR10] Bloetzer, C., Bovet, P., Suris, J. C., Simeoni, U., Paradis, G., & Chiolero, A. (2015). Screening for cardiovascular disease risk factors beginning in childhood. *Public Health Reviews,**36*, 9. 10.1186/s40985-015-0011-2 29450037 10.1186/s40985-015-0011-2 PMC5804494

[CR11] Borhanuddin, B., Mohd Nawi, A., Shah, S. A., Abdullah, N., Syed Zakaria, S. Z., Kamaruddin, M. A., Velu, C. S., Ismail, N., Abdullah, M. S., Ahmad Kamat, S., Awang, A., Hamid, M. A., & Jamal, R. (2018). 10-Year cardiovascular disease risk estimation based on lipid profile-based and BMI-based Framingham risk scores across multiple sociodemographic characteristics: The Malaysian Cohort Project. *The Scientific World Journal,**2018*, 2979206. 10.1155/2018/2979206 30111990 10.1155/2018/2979206 PMC6077456

[CR12] Brown, J. C., Gerhardt, T. E., & Kwon, E. (2022). *Risk factors for coronary artery disease*. StatPearls Publishing. 32119297

[CR13] Chan, Y. Y., Sahril, N., Rezali, M. S., Kuang Kuay, L., Baharudin, A., Abd Razak, M. A., Azlan Kassim, M. S., Mohd Yusoff, M. F., Omar, M. A., & Ahmad, N. A. (2021). Self-reported modifiable risk factors of cardiovascular disease among older adults in Malaysia: A cross-sectional study of prevalence and clustering. *International Journal of Environmental Research and Public Health,**18*(15), 7941. 10.3390/ijerph18157941 34360235 10.3390/ijerph18157941 PMC8345577

[CR14] Chee, H. P., Hazizi, A. S., Barakatun Nisak, M. Y., & Mohd Nasir, M. T. (2014a). Metabolic risk factors among government employees in Putrajaya, Malaysia. *Sains Malaysiana,**43*(8), 1165–1174.

[CR15] Chee, H. P., Hazizi, A. S., Barakatun Nisak, Y., & Mohd Nasir, M. T. (2014b). A randomised controlled trial of a Facebook-based physical activity intervention for government employees with metabolic syndrome. *Malaysian Journal of Nutrition,**20*(2), 165–181.

[CR16] Chee, H. P., Hazizi, A. S., Barakatun Nisak, Y., & Mohd Nasir, M. T. (2017). Effectiveness of physical activity intervention among government employees with metabolic syndrome. *Journal of Exercise Science and Fitness,**15*(2), 55–62. 10.1016/j.jesf.2017.07.003 29541133 10.1016/j.jesf.2017.07.003 PMC5812876

[CR17] Ching, Y. K., Chin, Y. S., Appukutty, M., Gan, W. Y., Ramanchadran, V., & Chan, Y. M. (2018). Prevalence of metabolic syndrome and its associated factors among vegetarians in Malaysia. *International Journal of Environmental Research and Public Health,**15*(9), 2031. 10.3390/ijerph15092031 30227682 10.3390/ijerph15092031 PMC6164423

[CR18] Chu, A. H., & Moy, F. M. (2013a). Joint association of sitting time and physical activity with metabolic risk factors among middle-aged Malays in a developing country: A cross-sectional study. *PLoS ONE,**8*(4), e61723. 10.1371/journal.pone.0061723 23613917 10.1371/journal.pone.0061723 PMC3629118

[CR19] Chu, A. H., & Moy, F. M. (2013b). Associations of occupational, transportation, household and leisure-time physical activity patterns with metabolic risk factors among middle-aged adults in a middle-income country. *Preventive Medicine,**57*(Suppl), S14–S17. 10.1016/j.ypmed.2012.12.011 23276774 10.1016/j.ypmed.2012.12.011

[CR20] Chu, A. H., & Moy, F. M. (2014). Association between physical activity and metabolic syndrome among Malay adults in a developing country, Malaysia. *Journal of Science and Medicine in Sport,**17*(2), 195–200. 10.1016/j.jsams.2013.04.003 23665093 10.1016/j.jsams.2013.04.003

[CR21] Department of Statistics Malaysia. (2021). *Statistics on causes of death, Malaysia, 2021*. Prime Minister’s Department, Putrajaya.

[CR22] Department of Statistics Malaysia. (2022). *Statistics on causes of death, Malaysia, 2022*. Prime Minister’s Department, Putrajaya.

[CR23] Gandhi, S., Chen, S., Hong, L., Sun, K., Gong, E., Li, C., Yan, L. L., & Schwalm, J. D. (2017). Effect of mobile health interventions on the secondary prevention of cardiovascular disease: Systematic review and meta-analysis. *Canadian Journal of Cardiology,**33*(2), 219–231. 10.1016/j.cjca.2016.08.017 27956043 10.1016/j.cjca.2016.08.017

[CR24] Hackshaw, A., Morris, J. K., Boniface, S., Tang, J. L., & Milenković, D. (2018). Low cigarette consumption and risk of coronary heart disease and stroke: Meta-analysis of 141 cohort studies in 55 study reports. *BMJ,**360*, j5855. 10.1136/bmj.j5855 29367388 10.1136/bmj.j5855 PMC5781309

[CR25] Hasbullah, F. Y., Mohd Yusof, B. N., Abdul Ghani, R., Mat Daud, Z., Appannah, G., Abas, F., & Shyam, S. (2022). Maternal and dietary factors are associated with metabolic syndrome in women with a previous history of gestational diabetes mellitus. *International Journal of Environmental Research and Public Health,**19*(24), 16797. 10.3390/ijerph192416797 36554678 10.3390/ijerph192416797 PMC9779785

[CR26] Hassan, I. I., Muhmmad, I. N., Ismail, S. B., Kadir, A. A., Mohd Noor, N., & Nik Hussain, N. H. (2017). Effect of health education program on cardiovascular risk factors among menopausal women in Malaysia. *International Medical Journal,**24*(1), 51–55.

[CR27] Institute for Public Health. (2019). *Non-communicable diseases, healthcare demand, and health literacy: Key findings.* National Health and Morbidity Survey (NHMS) 2019.

[CR28] Iqbal, S. P., Ramadas, A., Fatt, Q. K., Shin, H. L., Onn, W. Y., & Kadir, K. A. (2020). Relationship of sociodemographic and lifestyle factors and diet habits with metabolic syndrome (MetS) among three ethnic groups of the Malaysian population. *PLoS ONE,**15*(3), e0224054. 10.1371/journal.pone.0224054 32191727 10.1371/journal.pone.0224054 PMC7082049

[CR29] Johari, S. M., & Shahar, S. (2014). Metabolic syndrome: The association of obesity and unhealthy lifestyle among Malaysian elderly people. *Archives of Gerontology and Geriatrics,**59*(2), 360–366. 10.1016/j.archger.2014.04.003 24882592 10.1016/j.archger.2014.04.003

[CR30] Juhari, S. N., Mohd Yusoff, S. S., Badrin, S., & Tengku Ismail, T. A. (2020). Evaluation of cardiovascular disease assessment and preventive activities behaviour among the female nurses. *International Medical Journal,**27*(6), 661–665.

[CR31] Kuan, P. X., Chan, W. K., Chua, P. F., Yeo, J., Sapri, F. E., Bujang, M. A., & Said, A. (2020). Lifestyle factors associated with cardiovascular risk among healthcare workers from the tertiary hospitals in Sarawak. *Malaysian Family Physician,**15*(1), 15–22. 32284800 PMC7136671

[CR32] Lee, I., Kim, S., & Kang, H. (2019). Lifestyle risk factors and all-cause and cardiovascular disease mortality: Data from the Korean Longitudinal Study of Aging. *International Journal of Environmental Research and Public Health,**16*(17), 3040. 10.3390/ijerph16173040 31443353 10.3390/ijerph16173040 PMC6747152

[CR33] Lee, S. C., Moy, F. M., Sii, H. L., & Hairi, N. N. (2022). The association of psychosocial health with metabolic syndrome among school teachers in the state of Malacca. *Journal of Health and Translational Medicine,**25*(2), 7–14.

[CR34] Lim, C. W., Kasim, S., Ismail, J. R., Chua, N. Y., Najme Khir, R., Zainal Abidin, H. A., Abdul Rahman, E., Mohd Arshad, M. K., Ibrahim Othman, Z., & Yusoff, K. (2016). Prevalence of atrial fibrillation in the Malaysian communities. *Heart Asia,**8*(2), 62–66. 10.1136/heartasia-2016-010775 27933105 10.1136/heartasia-2016-010775 PMC5133392

[CR35] Lim, Y. C., Hoe, V. C. W., Darus, A., & Bhoo-Pathy, N. (2018). Association between night-shift work, sleep quality and metabolic syndrome. *Occupational and Environmental Medicine,**75*(10), 716–723. 10.1136/oemed-2018-105104 30032104 10.1136/oemed-2018-105104

[CR36] Lopez, E. O., Ballard, B. D., & Jan, A. (2022). *Cardiovascular disease*. StatPearls Publishing. 30571040

[CR37] Mahadzir, M. D. A., Quek, K. F., & Ramadas, A. (2020). Nutrition and lifestyle behavior peer support program for adults with metabolic syndrome: Outcomes and lessons learned from a feasibility trial. *Nutrients,**12*(4), 1091. 10.3390/nu12041091 32326541 10.3390/nu12041091 PMC7230344

[CR38] Manaf, M. R. A., Nawi, A. M., Tauhid, N. M., Othman, H., Rahman, M. R. A., Yusoff, H. M., Safian, N., Ng, P. Y., Manaf, Z. A., Kadir, N. B. A., Yanasegaran, K., Basir, S. M. A., Ramakrishnappa, S., & Ganasegeran, K. (2021). Prevalence of metabolic syndrome and its associated risk factors among staffs in a Malaysian public university. *Scientific Reports,**11*(1), 8132. 10.1038/s41598-021-87248-1 33854087 10.1038/s41598-021-87248-1 PMC8047014

[CR39] Ministry of Health Malaysia. (2017). *Clinical practice guidelines on primary and secondary prevention of cardiovascular disease*. Ministry of Health Malaysia.

[CR40] Mohd Noor, N., Sharifah Amirah, A. H., & Lili Husniati, Y. (2013). Cardiovascular risk: Associated factors, assessment and agreement between WHO/ISH risk prediction chart and Framingham scoring system among primary care patients. *International Journal of Collaborative Research on Internal Medicine and Public Health,**5*(12), 652.

[CR41] Omar, A., Husain, M. N., Jamil, A. T., Nor, N. S. M., Ambak, R., Fazliana, M., Zamri, N. L. A., & Aris, T. (2018). Effect of physical activity on fasting blood glucose and lipid profile among low income housewives in the MyBFF@home study. *BMC Women’s Health,**18*(Suppl 1), 103. 10.1186/s12905-018-0598-9 30066645 10.1186/s12905-018-0598-9 PMC6069292

[CR42] Omar, N. O., Ahmad, R. A., Mohd Shah, M. S., Aminuddin, A. A., & Chellappan, K. C. (2021). Amelioration of inflammation in young men with cardiovascular risks participating pedometer-based walking programme. *Medical Journal of Malaysia,**76*(3), 375–381. 34031337

[CR43] Ostman, C., Smart, N. A., Morcos, D., Duller, A., Ridley, W., & Jewiss, D. (2017). The effect of exercise training on clinical outcomes in patients with the metabolic syndrome: A systematic review and meta-analysis. *Cardiovascular Diabetology,**16*(1), 110. 10.1186/s12933-017-0590-y 28854979 10.1186/s12933-017-0590-y PMC5577843

[CR44] Pan, A., Wang, Y., Talaei, M., & Hu, F. B. (2015). Relation of smoking with total mortality and cardiovascular events among patients with diabetes mellitus: A meta-analysis and systematic review. *Circulation,**132*(19), 1795–1804. 10.1161/CIRCULATIONAHA.115.017926 26311724 10.1161/CIRCULATIONAHA.115.017926 PMC4643392

[CR45] Paudel, S., Owen, A. J., Owusu-Addo, E., & Smith, B. J. (2019). Physical activity participation and the risk of chronic diseases among South Asian adults: A systematic review and meta-analysis. *Scientific Reports,**9*(1), 9771. 10.1038/s41598-019-46154-3 31278314 10.1038/s41598-019-46154-3 PMC6611898

[CR46] Peters, M. D. J., Godfrey, C. M., Khalil, H., McInerney, P., Parker, D., & Cassia Baldini Soares, C. B. (2015). Guidance for conducting systematic scoping reviews. *International Journal of Evidence-Based Healthcare,**13*(3), 141–146. 10.1097/XEB.0000000000000050 26134548 10.1097/XEB.0000000000000050

[CR47] Rahmati Najarkolaei, F., Ghaffarpasand, E., Gholami Fesharaki, M., & Jonaidi Jafari, N. (2015). Nutrition and physical activity educational intervention on CHD risk factors: A systematic review study. *Archives of Iranian Medicine,**18*(1), 51–57. 25556387

[CR48] Rashid, N. A., Nawi, A. M., & Khadijah, S. (2019). Exploratory analysis of traditional risk factors of ischemic heart disease (IHD) among predominantly Malay Malaysian women. *BMC Public Health,**19*(Suppl 4), 545. 10.1186/s12889-019-6855-5 31196022 10.1186/s12889-019-6855-5 PMC6565533

[CR49] Rasiah, R., Thangiah, G., Yusoff, K., Manikam, R., Chandrasekaran, S. K., Mustafa, R., & Bakar, N. B. (2015). The impact of physical activity on cumulative cardiovascular disease risk factors among Malaysian adults. *BMC Public Health,**15*, 1242. 10.1186/s12889-015-2577-5 26673166 10.1186/s12889-015-2577-5 PMC4681048

[CR50] Shah, S. A., Lee, J., Khalid, M. S., Najid, F., Haniff, I. S., & Ghazi, A. M. (2015). Prevalence and risk factors of premature coronary artery disease: A comparative cross-sectional study between two time frames in Malaysia. *Malaysian Journal of Public Health Medicine,**15*(3), 157–166.

[CR51] Shahar, S., Adznam, S. N., Lee, L. K., Yusof, N. A., Salleh, M., & Mohamed Sakian, N. I. (2013). A nutrition education intervention for anthropometric and biochemical profiles of rural older Malays with metabolic syndrome. *Public Health Nursing,**30*(2), 140–149. 10.1111/j.1525-1446.2012.01051.x 23452108 10.1111/j.1525-1446.2012.01051.x

[CR52] Shariff, S. G., Sooryanarayana, R., Ho, B. K., Omar, M. A., Krishnapillai, A. D., Mohd Tohit, N., Inche Zainal Abidin, S., Ariaratnam, S., & Ahmad, N. A. (2020). Cardiovascular disease risk factors among older people: Data from the National Health and Morbidity Survey 2015. *PLoS ONE,**15*(10), e0240826. 10.1371/journal.pone.0240826 33085718 10.1371/journal.pone.0240826 PMC7577487

[CR53] Torquati, L., Mielke, G. I., Brown, W. J., & Kolbe-Alexander, T. (2018). Shift work and the risk of cardiovascular disease. A systematic review and meta-analysis including dose–response relationship. *Scandinavian Journal of Work, Environment and Health,**44*(3), 229–238. 10.5271/sjweh.3700 29247501 10.5271/sjweh.3700

[CR54] Tricco, A. C., Lillie, E., Zarin, W., O’Brien, K. K., Colquhoun, H., Levac, D., Moher, D., Peters, M. D. J., Horsley, T., Weeks, L., Hempel, S., Akl, E. A., Chang, C., McGowan, J., Stewart, L., Hartling, L., Aldcroft, A., Wilson, M. G., Garritty, C., … Straus, S. E. (2018). PRISMA extension for scoping reviews (PRISMA-ScR): Checklist and explanation. *Annals of Internal Medicine,**169*(7), 467–473. 10.7326/M18-0850 30178033 10.7326/M18-0850

[CR55] Van Hecke, A., Heinen, M., Fernández-Ortega, P., Graue, M., Hendriks, J. M., Høy, B., Köpke, S., Lithner, M., & Van Gaal, B. G. (2017). Systematic literature review on effectiveness of self-management support interventions in patients with chronic conditions and low socio-economic status. *Journal of Advanced Nursing,**73*(4), 775–793. 10.1111/jan.13159 27653960 10.1111/jan.13159

[CR56] Veritas Health Innovation. (2022). *Covidence systematic review software*. Available: https://www.covidence.org

[CR57] Wan Musa, W. Z., Ahmad, A., Abu Bakar, N. Z. F., Wan-Arfah, N., Ramli, A. W., & Naing, N. N. (2022). Predictors of coronary heart disease (CHD) among Malaysian adults: Findings from MyDiet-CHD Study. *Malaysian Journal of Medicine and Health Sciences,**18*(6), 259–269. 10.47836/mjmhs18.6.34 10.47836/mjmhs18.6.34

[CR58] World Health Organization. (2021). *Cardiovascular diseases (CVDs)*. Available: https://www.who.int/news-room/fact-sheets/detail/cardiovascular-diseases-(cvds)

[CR59] Yaacob, N. M., Yaacob, N. A., Ismail, A. A., Che Soh, N. A. A., Ismail, M. S., Mohamed, H. J. J., & Hairon, S. M. (2016). Dumbbells and ankle-wrist weight training leads to changes in body composition and anthropometric parameters with potential cardiovascular disease risk reduction. *Journal of Taibah University Medical Sciences,**11*(5), 439–447. 10.1016/j.jtumed.2016.06.005

[CR60] Yeow, T. P., Khir, A. S., Ismail, A. A., Ismail, I. S., Kamarul Imran, M., Khalid, B. A., Kamaruddin, N. A., Azwany, Y. N., Mustafa, N., Osman, A., Md Isa, S. H., Bebakar, W. M., & Nazaimoon, W. M. (2012). Predictors of ischaemic heart disease in a Malaysian population with the metabolic syndrome. *Diabetic Medicine,**29*(11), 1378–1384. 10.1111/j.1464-5491.2012.03741.x 22803824 10.1111/j.1464-5491.2012.03741.x

[CR61] Zhang, X., Liu, Y., Li, S., Lichtenstein, A. H., Chen, S., Na, M., Veldheer, S., Xing, A., Wang, Y., Wu, S., & Gao, X. (2021). Alcohol consumption and risk of cardiovascular disease, cancer and mortality: A prospective cohort study. *Nutrition Journal.,**20*, 13. 10.1186/s12937-021-00671-y 33522924 10.1186/s12937-021-00671-y PMC7852289

