# Supplementary material for: Lifestyle-Related Risk Factors and Primary Prevention Strategies for Cardiovascular Diseases in a Middle-Income Country: A Scoping Review and Implication for Future Research
Source: J Prev (2022). 2024 Jun 5;45(4):579–609. doi: 10.1007/s10935-024-00782-2 (PMC11271377; doi:10.1007/s10935-024-00782-2)
Supplement: Supplementary file 1 — Supplementary file1 (DOCX 16 KB) [file 10935_2024_782_MOESM1_ESM.docx]

**Supplementary Table S1: Search strategy for Ovid Medline**

| **No.** | **Searches** | **Results** |
| --- | --- | --- |
| 1 | cardiovascular.mp. or Cardiovascular Diseases/ | 625099 |
| 2 | coronary disease*.mp. or Coronary Disease/ | 141252 |
| 3 | heart disease*.mp. or Heart Diseases/ | 256473 |
| 4 | CVD.mp. | 40561 |
| 5 | Cardiac.mp. | 813178 |
| 6 | Framingham.mp. | 9550 |
| 7 | metabolic syndrome.mp. or Metabolic Syndrome/ | 60792 |
| 8 | lifestyle.mp. or Life Style/ | 139173 |
| 9 | nutrition.mp. | 252304 |
| 10 | diet*mp. Or Diet/ | 807779 |
| 11 | physical activit*mp. Or Exercise/ | 218010 |
| 12 | sleep.mp. or Sleep/ | 216549 |
| 13 | Smoking.mp. or Smoking/ or Cigarette Smoking/ or Tobacco Smoking/ | 301532 |
| 14 | behavio*.mp. | 1707838 |
| 15 | Malaysia.mp or Malaysia/ | 22983 |
| 16 | 1 or 2 or 3 or 4 or 5 or 6 or 7 | 1566506 |
| 17 | 8 or 9 or 10 or 11 or 12 or 13 or 14 | 3133751 |
| 18 | 15 and 16 and 17 | 264 |
| 19 | Limit 18 to (yr=”2012-Current” and “all adult (19 plus years)”) | 127 |
